# A Multifunctional Bimetallic Nanoplatform for Synergic Local Hyperthermia and Chemotherapy Targeting HER2‐Positive Breast Cancer

**DOI:** 10.1002/advs.202308316

**Published:** 2024-02-21

**Authors:** Li Zhao, Fei Chang, Yao Tong, Jiawei Yin, Jiawen Xu, Hui Li, Lutao Du, Yanyan Jiang

**Affiliations:** ^1^ Liquid‐Solid Structural Evolution & Processing of Materials (Ministry of Education) School of Materials Science and Engineering Shandong University Jinan Shandong 250061 China; ^2^ The Second Hospital of Shandong University Jinan Shandong 250033 China; ^3^ Department of Pathology Shandong Provincial Hospital affiliated to Shandong First Medical University Jinan Shandong 250021 China; ^4^ Department of Clinical Laboratory Qilu Hospital of Shandong University Jinan Shandong 250012 China; ^5^ Shandong Provincial Key Laboratory of Innovation Technology in Laboratory Medicine Jinan Shandong 250033 China; ^6^ Shandong Provincial Clinical Medicine Research Center for Clinical Laboratory Jinan Shandong 250033 China

**Keywords:** bimetallic AuAg HNSs, chemo‐phototherapy, HER2‐positive breast cancer, nanotherapeutic platform, pyrotinib

## Abstract

Anti‐HER2 (human epidermal growth factor receptor 2) therapies significantly increase the overall survival of patients with HER2‐positive breast cancer. Unfortunately, a large fraction of patients may develop primary or acquired resistance. Further, a multidrug combination used to prevent this in the clinic places a significant burden on patients. To address this issue, this work develops a nanotherapeutic platform that incorporates bimetallic gold‐silver hollow nanoshells (AuAg HNSs) with exceptional near‐infrared (NIR) absorption capability, the small‐molecule tyrosine kinase inhibitor pyrotinib (PYR), and Herceptin (HCT). This platform realizes targeted delivery of multiple therapeutic effects, including chemo‐and photothermal activities, oxidative stress, and immune response. In vitro assays reveal that the HCT‐modified nanoparticles exhibit specific recognition ability and effective internalization by cells. The released PYR inhibit cell proliferation by downregulating HER2 and its associated pathways. NIR laser application induces a photothermal effect and tumor cell apoptosis, whereas an intracellular reactive oxygen species burst amplifies oxidative stress and triggers cancer cell ferroptosis. Importantly, this multimodal therapy also promotes the upregulation of genes related to TNF and NF‐κB signaling pathways, enhancing immune activation and immunogenic cell death. In vivo studies confirm a significant reduction in tumor volume after treatment, substantiating the potential effectiveness of these nanocarriers.

## Graphical abstract

A nanotherapeutic platform targeting HER2‐positive breast cancer is developed, consisting of bimetallic gold‐silver hollow nanoshells with excellent near‐infrared absorption. These nanoshells are loaded with the small‐molecule tyrosine kinase inhibitor pyrotinib and surface‐modified with Herceptin. This platform realizes targeted delivery of multiple therapeutic effects, including chemo‐ and photothermal activities, oxidative stress, and immune response.

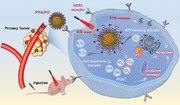

## 1 Introduction

Breast cancer is the most common malignancy among women, with ≈20–25% cases characterized by human epidermal growth factor receptor 2 (HER2) overexpression.^[^ ^1^, ^2^ ^]^ This subtype of breast cancer is associated with high malignancy, propensity for recurrence, distant metastasis, and poor prognosis.^[^ ^3^ ^]^ Monoclonal antibody drugs, such as trastuzumab and pertuzumab, have notably improved the prognosis of patients with HER2‐positive breast cancer.^[^ ^4^, ^5^ ^]^ However, the development of resistance and disease progression are common, and the high doses and long treatment durations significantly affect quality of life among patients.^[^ ^6^, ^7^ ^]^

Pyrotinib (PYR) is a small molecule and an irreversible pan‐HER tyrosine kinase inhibitor (TKI) that targets receptor TKI to achieve permanent receptor inactivation. PYR realized this by alkylating cysteine residues within the intracellular ATP‐binding domain of the receptor.^[^ ^8^, ^9^ ^]^ Compared to large‐molecule monoclonal antibody drugs, small‐molecule TKIs are more likely to cross the blood‐brain barrier. In clinical research, PYR has demonstrated effective improvements in disease‐free and overall survival in patients with HER2‐positive breast cancer.^[^ ^10^ ^]^ Nonetheless, the free form of this small‐molecule chemotherapeutic agent has limitations such as poor bioavailability and short action period, and is associated with systemic and organ toxicity. As an alternative approach, nanoparticle‐based drug delivery has gained increasing attention in breast cancer treatment.^[^ ^11^ ^]^ In recent years, numerous stimuli‐responsive nanocarriers have been developed to facilitate controlled release and sustained delivery of biologically active substances. These carriers are also used to implement specific novel therapies in tandem with external physical stimuli such as phototherapy, acoustic therapy, and magnetotherapy.^[^ ^12^, ^13^ ^]^

Noble metal‐based nanocarriers are among the most promising systems for this purpose because of their good biocompatibility, stability, and easily modified and adjustable localized surface plasmon resonance (LSPR) properties.^[^ ^14^, ^15^ ^]^ In particular, gold and silver hollow nanoshells (AuAg HNSs) demonstrate efficient drug‐loading capabilities due to their distinctive hollow interiors and porous wall structures. These nanoshells can be engineered to release drugs under specific stimuli such as pH or temperature changes.^[^ ^16^ ^]^ Furthermore, AuAg HNSs possess an exceptional near‐infrared (NIR) light absorption capacity. On the one hand, this property can be utilized for photothermal therapy of tumor cells. On the other hand, the surface plasmon resonance of AuAg HNSs under NIR light irradiation triggers the generation of hot electrons, thereby augmenting their catalytic activity. This enhancement fosters the production of reactive oxygen species (ROS), subsequently disrupting the homeostatic equilibrium of cells.^[^ ^17^, ^18^ ^]^

**Scheme 1:**
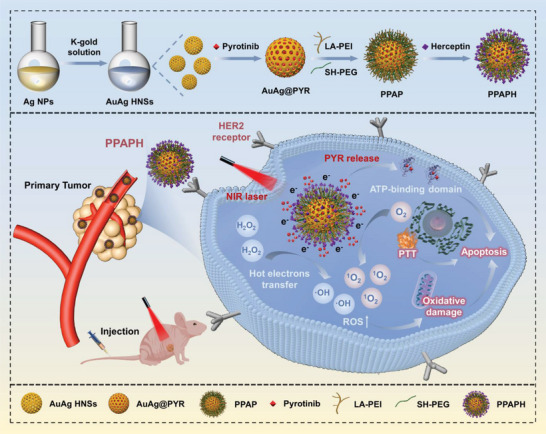
Synthetic procedure of PPAPH NPs and schematic diagram of PPAPH NPs targeted synergistic therapy for HER2 positive breast cancer.

In this study, we engineered a versatile gold‐silver hollow nanocarrier system for targeted multimodal synergistic therapy of HER2‐positive breast cancer (**Scheme** **1**). AuAg HNSs were synthesized using a simple electrodisplacement method, resulting in a unique hollow porous structure for the efficient loading of PYR. Subsequently, the nanoshell surfaces were modified with lipoic acid‐polyethyleneimine (LA‐PEI) and thiolated polyethylene glycol (SH‐PEG) to prevent premature drug release and enhance stability. The nanoparticles were further modified with Herceptin (HCT) to precisely target HER2‐overexpressing tumor cells. In vitro cell experiments demonstrated that the synergistic effect of localized laser‐induced hyperthermia and chemotherapy increased cytotoxicity and was highly in suppressing HER2 overexpressing BT474 cells. Sequencing analysis revealed that PYR downregulated the expression of HER2 members (epidermal growth factor receptor, HER2, and HER3) and their downstream signaling pathways (PIK3 and MAPK). Notably, intratumoral photocatalysis triggered by the AuAg HNSs induced a significant increase in ROS, thereby enhancing oxidative stress and promoting tumor cell death. These synergistic actions of the AuAg HNSs nanocarrier system resulted in a markedly superior therapeutic effect, significantly reducing the tumor size in a mouse model of HER2‐positive breast cancer.

## 2 Results and Discussion

**Figure 1:**
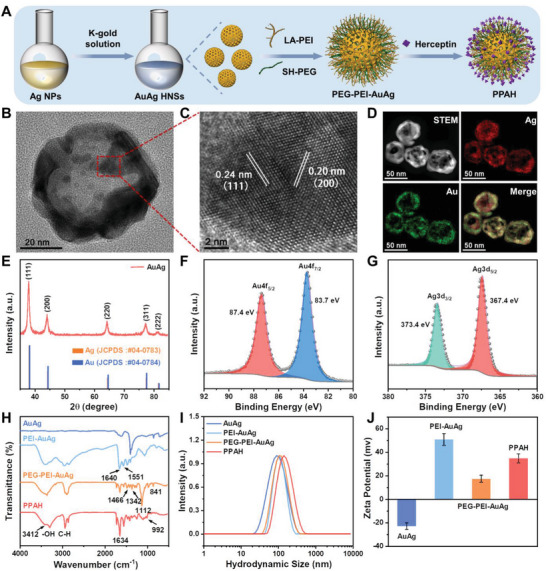
A) A scheme illustrating the synthesis process of PPAH NPs. B) TEM image of AuAg HNSs. C) HR‐TEM image of AuAg HNSs. D) Elemental mapping of AuAg HNSs. E) XRD pattern of AuAg HNSs. F,G) High‐resolution XPS spectra of Au 4f and Ag 3d. H) FTIR spectra of AuAg HNSs, PEI‐AuAg, PEG‐PEI‐AuAg, and PPAH. I,J) Hydrodynamic diameter and zeta potential of AuAg HNSs, PEI‐AuAg, PEG‐PEI‐AuAg, and PPAH (*n* = 3, mean ± s.d.).

The synthesis of PEG‐PEI‐AuAg@HCT (PPAH) NPs is shown in **Figure** **1**A. Silver nanoparticles (Ag NPs) were produced by the chemical reduction of AgNO_3_ with sodium citrate and ascorbic acid. As shown in the transmission electron microscopy (TEM) images (Figure S1A,B, Supporting Information), the synthesized Ag NPs are relatively uniform spheres, ≈47 nm in diameter, with a surface plasmon resonance (SPR) peak at 420 nm. After mixing with HAuCl_4_, the color of the solution changed from yellow to blue and a single SPR peak appeared at ≈800 nm, which confirmed the formation of AuAg HNSs (Figure S2, Supporting Information). These AuAg HNSs had a size of 52 ± 0.8 nm, and TEM imaging revealed a darker shell and brighter inner cavities, further confirming the formation of AuAg hollow nanostructures (Figure S1C,D, Supporting Information). Furthermore, the SPR absorption of AuAg HNSs was adjusted by modifying the ratio of HAuCl_4_ to Ag NPs (Figure S3, Supporting Information). High‐resolution TEM (HR‐TEM) images of the shells of AuAg HNSs (Figure 1B,C) exhibited lattice fringes of 0.24 and 0.20 nm, corresponding to the (111) and (200) planes of the hexagonal phase of Au or Ag crystals, respectively. Elemental mapping (Figure 1D and Figure S4, Supporting Information) showed the colocalization of Au and Ag, confirming that the dark shell observed under TEM was characteristic of the hybrid Ag/Au. X‐ray diffraction (XRD) analysis of the AuAg HNSs revealed distinct peaks at 37.94°, 44.18°, 64.52°, 77.48°, and 81.6° (Figure 1E), corresponding to the (111), (200), (220), (311), and (222) planes, respectively. These diffraction patterns were unequivocally indexed to face‐centered cubic Ag (JCPDS 04–0783) and Au (JCPDS 04–0784). X‐ray photoelectron spectrometer (XPS) spectra indicated the co‐existence of Au^0^ and Pt^0^ in the AuAg HNSs (Figure S5, Supporting Information and Figure 1F,G), further validating the alloy nature of the nanoparticles. It is worth emphasizing that previous studies have shown that such hollow nanostructures are porous, which allows the diffusion of reactants (Ag, H, etc.) and water to the interior of the nanoparticles.^[^ ^19^, ^20^ ^]^

To obtain stable nanoparticles with targeting capabilities, the surfaces of the AuAg HNSs were further modified and functionalized. LA‐PEI was synthesized using a simple amide coupling method and grafted onto the surface of AuAg HNSs via Au‐S bonds. Similarly, SH‐PEG adhered to the PEI‐AuAg surface, forming PEG‐PEI‐AuAg (PPA). HCT was subsequently immobilized on the surface of PEG‐PEI‐AuAg with the formation of an amide bond between the carboxyl group of HCT and the amino group of LA‐PEI, resulting in the formation of nanocomposite PPAH. The formation of the nanocomposite was verified using Fourier transform infrared (FTIR) spectroscopy (Figure 1H). The FTIR spectrum of PEI‐AuAg displayed a peak at 1640 cm^−1^, corresponding to the stretching vibration of the carbon‐oxygen double bond in the amide group. Additionally, a peak at 1551 cm^−1^ represented the overlapping bending modes in the N─H plane and the C─N stretching vibration in PEI. The absorption peaks at 2887 and 1710 cm^−1^, attributable to the C─H and C═O stretching vibrations in the PEG molecule, provided strong evidence for successful PEGylation. The transmission bands of PPAH at 1636, 1540, and 3286 cm^−1^ could be assigned to the amide I, amide II, and amine N─H. A band at 992 cm^−1^ corresponded to the characteristic absorption of HCT (Figure S6, Supporting Information), confirming the successful coupling of HCT. Dynamic light scattering (DLS) analysis further confirmed the formation of the nanocomposites (Figure 1I,J). As a result of PEI grafting onto the surface of AuAg HNSs, the hydrodynamic diameter of the PEI‐AuAg increased to 106 nm from the original 91 nm, and the zeta potential changed from −24 to +52 mV due to PEI surface modification. After modification with HCT, the hydrodynamic diameter of PPAH increased to 142 nm and its zeta potential decreased to 37 mV, indicating a successful conjugation reaction. Figure S7, Supporting Information, confirms the uniform polymeric corona of ≈5 nm on the AuAg HNSs. Moreover, PPAH NPs with good colloidal stability maintained excellent dispersity in water, PBS, and DMEM for 7 days, and the hydrodynamic diameter did not exhibit any discernible changes (Figure S8, Supporting Information), indicating their potential for use in biological experiments.

**Figure 2:**
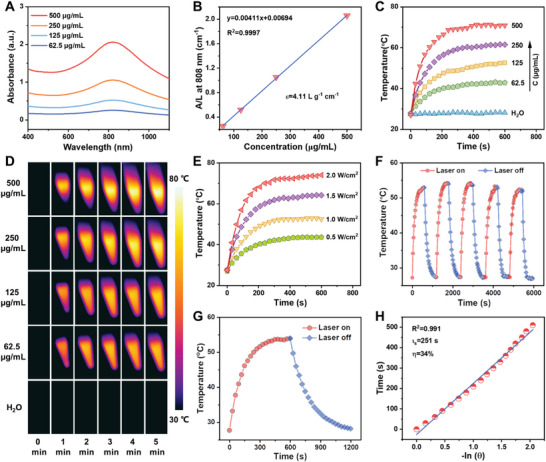
A) UV–vis‐NIR absorption spectra of PPAH NPs at different concentrations. B) The mass extinction coefficient of PPAH NPs at 808 nm. C) Temperature evaluation curves and D) corresponding infrared thermal images of PPAH NPs with different concentrations under 808 nm laser irradiation at 1.0 W cm^−2^. E) Temperature elevation curves of PPAH NPs (125 µg mL^−1^) under 808 nm laser irradiation at different power densities. F) Photothermal curves of PPAH NPs (125 µg mL^−1^) for five heating/cooling cycles under 808 nm laser irradiation. G) Temperature elevation curve of PPAH NPs (125 µg mL^−1^) under 808 nm laser irradiation and cooling curve after the laser power off. H) Linear relationship between time and ‐ln θ calculated from a cooling period in (G).

PPAH NPs exhibited notable absorption in the NIR region (**Figure** **2**A). The extinction coefficient (ε) of PPAH was calculated to be 4.11 L g^−1^ cm^−1^ at 808 nm according to the Lambert‐Beer law (Figure 2B), revealing that PPAH NPs could serve as highly effective photothermal conversion reagent.^[^ ^21^, ^22^ ^]^ To evaluate their photothermal performance, an aqueous PPAH solution was subjected to an NIR laser for 10 min. Temperature variations and corresponding images were captured using an infrared thermographic camera. The results showed that the photothermal effect of PPAH NPs was concentration‐dependent, as the temperature could be precisely regulated between 28.1 and 71.0 °C by adjusting the nanoparticle concentration. However, under the same conditions, the temperature elevation detected in the water was negligible (Figure 2C,D). Moreover, the temperature elevation patterns of PPAH NPs depended on the laser power density (Figure 2E). When a dispersion of 125 µg mL^−1^ nanoparticles was exposed to 808 nm laser irradiation at 2.0 W cm^−2^ for 10 min, the temperature increased to 74.0 °C. Importantly, no notable temperature change was observed during the five on/off cycles of irradiation (Figure 2F), suggesting that the PPAH NPs have satisfactory photothermal stability. Finally, the photothermal conversion efficiency (PCE) was calculated to be 34% (Figure 2G,H), demonstrating the high potential of these NPs as photothermal agents.

**Figure 3:**
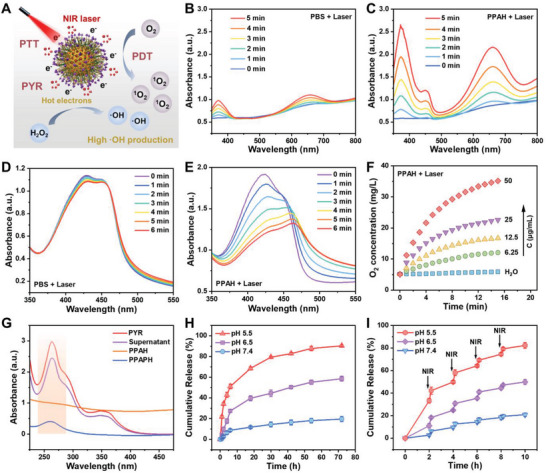
A) Schematic diagram of ROS production and drug release of PPAPH NPs under NIR laser irradiation. UV–vis absorption spectra of TMB solution containing B) PBS and C) PPAH NPs upon exposure to NIR laser irradiation. UV–vis absorption spectra of DPBF solution containing D) PBS and E) PPAH NPs upon exposure to NIR laser irradiation. F) O_2_ generation in H_2_O_2_ solution (10 mm) with different concentrations of PPAH NPs with irradiation. G) UV–vis absorption spectra of PYR, supernatant, PPAH, and PPAPH. H) Cumulative release of PYR from PPAPH in PBS at pH 7.4, 6.5 and 5.5 (*n* = 3, mean ± s.d.). (I) Laser triggered release of PYR from PPAPH in PBS at pH 7.4, 6.5 and 5.5 (*n* = 3, mean ± s.d.).

The catalytic activity of PPAH NPs was subsequently evaluated (**Figure** **3**A). 3,3′,5,5′‐tetramethylbenzidine (TMB) was used as a probe to assess the peroxidase‐like catalytic activity of PPAH NPs. In an acidic environment, these NPs reacted with H_2_O_2_ to produce oxidized hydroxyl radicals (•OH), which prompted the oxidation of TMB, resulting in the formation of blue‐colored oxTMB with characteristic absorbances at 370 and 652 nm (Figure S9, Supporting Information). The catalytic activity of PPAH NPs was concentration‐dependent (Figure S10, Supporting Information). After NIR light application, a pronounced increase was observed in the absorbance at 652 nm, indicating a substantial generation of •OH. This implies that the peroxidase activity of the PPAH NPs was amplified by light (Figure 3B,C). We also employed 1, 3‐diphenylisobenzofuran (DPBF) to monitor the production of singlet oxygen (^1^O_2_). The rapid degradation of DPBF under laser irradiation substantiated the photodynamic effect of PPAH NPs (Figure 3D,E, and Figure S11, Supporting Information). Additionally, the catalase‐mimetic activity of PPAH NPs was effectively enhanced by NIR irradiation. As indicated in Figure 3F and Figure S12, Supporting Information, when H_2_O_2_ was present, the oxygen production of the NIR‐irradiated system increased twofold compared to that of the control without NIR laser irradiation. These results underscore the augmented catalytic activity of PPAH NPs upon exposure to NIR irradiation, a phenomenon that may be ascribed to a plasma‐driven hot‐carrier reaction. Further investigations were conducted by measuring the photoluminescence (PL) of the AuAg HNSs at the single‐particle level. As shown in Figure S13, Supporting Information, the PL intensity of the AuAg HNSs immersed in water was almost completely quenched compared to that of the same single NPs exposed to air, revealing that the electronic charge was somehow exchanged between the nanoparticles and molecules.

By utilizing the hollow structure of the AuAg HNSs and the coating capability of PEI, we successfully loaded the hydrophobic drug PYR. Figure 3G shows the UV–vis absorbance spectra of various formulations. The characteristic absorption peaks of PYR were observed in PEG‐PEI‐AuAg@PYR@HCT (PPAPH), confirming successful PYR loading. The encapsulation efficiency (EE) and loading capacity (LC) were calculated to be 18.6% and 4.7%, respectively, based on the absorbance difference of PYR at 262 nm and the corresponding standard calibration curve (Figure S14, Supporting Information), using a method described in previous reports.^[^ ^23^ ^]^ We studied the drug‐release behavior of the nanocarriers by simulating the microenvironments of normal tissues, tumors and lysosomes with PBS buffer at pH 7.4, 6.5, and 5.5. The cumulative release of PYR in the buffer (pH 7.4) was 12% over 24 h, which increased to 40% when the pH was reduced to 6.5 and reached more than 70% when the pH was decreased to 5.5 (Figure 3H). This increase was attributed to weakened electrostatic interactions between PPAH and PYR in the acidic environment. The influence of NIR laser irradiation on the drug release behavior was evaluated under identical experimental conditions. As shown in Figure 3I, a sudden release of PYR was observed upon laser irradiation of the nanocarrier solution. In PBS solution at pH 5.5, PPAPH was applied NIR laser four times over a period of 10 h and finally 80% of PYR was released. We proposed that the photothermal effect generated by NIR laser irradiation of PPAPH NPs resulted in heat accumulation. The accumulated heat accelerated the movement of drug molecules, which led to the rapid release of PYR from the nanoparticles.^[^ ^24^, ^25^ ^]^ These results demonstrate that PPAPH NPs exhibit pH‐ and laser‐responsive characteristics, thereby minimizing drug leakage under physiological conditions while promoting drug release in acidic tumor microenvironments and under laser irradiation. Consequently, this design enables site‐specific drug release at the tumor and avoids the adverse effects of nanomedicines on the surrounding tissues and organs.

**Figure 4:**
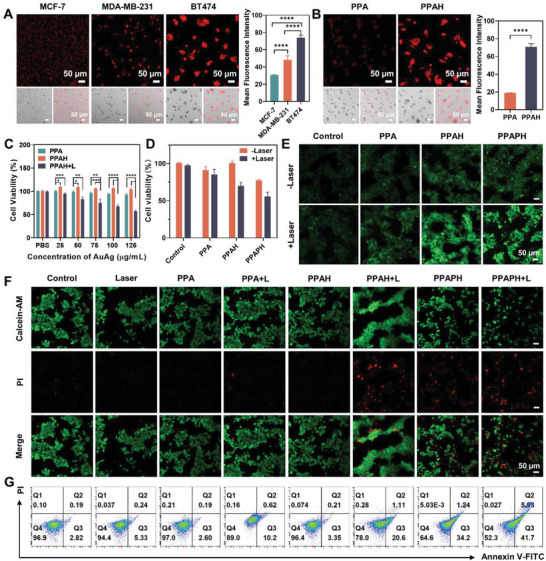
A) CLSM images of MDA‐MB‐231, MCF‐7, and BT474 cells after being treated with Cy5.5‐labeled PPAH NPs, and their corresponding mean fluorescence intensity analysis (*n* = 3, mean ± s.d.). B) CLSM images of BT474 cells incubated with the Cy5.5‐labeled PPA NPs and Cy5.5‐labeled PPAH NPs, and their corresponding mean fluorescence intensity analysis (*n* = 3, mean ± s.d.). Scale bar: 50 µm. C) Relative cell viabilities of BT474 cells treated with different concentrations (PBS, 25, 50, 75, 100, and 125 µg mL^−1^) of PPA, PPAH, or PPAH+L (*n* = 3, mean ± s.d.). The p values were calculated by one‐way ANOVA, ****p < 0.0001, ***p < 0.001, **p < 0.01, and *p < 0.05. D) Relative cell viability of BT474 cells after incubation with PBS, PPA, PPAH, and PPAPH with and without 808 nm laser irradiation (*n* = 3, mean ± s.d.). E) Intracellular ROS level of BT474 cells after different treatments. F) Calcein AM/PI staining of BT474 cells after different treatments. Scale bar: 50 µm. G) Flow cytometric analysis of BT474 cancer cells after different treatments.

We investigated the cellular uptake of PPAH NPs using confocal laser scanning microscopy (CLSM) with BT474 cells as a HER2‐positive model. The PPAH NPs were labeled with Cy5.5 fluorophore to monitor their intracellular localization. As shown in Figure S15, Supporting Information, the uptake of PPAH NPs increased with increasing incubation time, indicating efficient internalization by the tumor cells. The cell‐targeting behavior of PPAH NPs was further assessed using CLSM for MCF‐7 (HER2 negative), MDA‐MB‐231 (HER2 negative), and BT474 (HER2 positive) cells. As illustrated in **Figure** **4**A, the red fluorescence signal in BT474 cells was significantly stronger than those in MCF‐7 and 231 cells after 6 h of incubation. This difference was attributed to the specific binding of the highly expressed HER2 receptor in BT474 cells to more Cy5.5‐labeled PPAH NPs. To further assess the contribution of HCT‐modified NPs to the targeting of cellular uptake efficiency, PPA and PPAH NPs were labeled with Cy5.5. Confocal imaging revealed strong red fluorescent signals in cells cultured with the targeted PPAH NPs, whereas weaker fluorescent signals were observed in cells cultured with non‐targeted PPA NPs (Figure 4B). These results suggest that HCT surface modification significantly enhances the efficiency of targeted cellular uptake of nanoparticles.^[^ ^26^ ^]^ In addition, timely lysosomal escape is crucial for maintaining the pharmacological activity of endocytosed nanomedicines.^[^ ^27^ ^]^ To assess the lysosomal escape behavior of Cy5.5‐labeled PPAH NPs, lysosomes within BT474 cells were labeled with Lysotracker, and fluorescence imaging was conducted using CLSM (Figure S16, Supporting Information). The escape rate was quantified through Pearson confocal analysis. Figure S17, Supporting Information showed the Pearson correlation coefficient between the green signal of Cy5.5 and the red signal of the Lysotracker as a function of incubation time. The coefficient at 4 h post‐treatment exceeded that at 2 h, suggesting that PPAH NPs had not yet been sufficiently internalized by the cells at the earlier time point. With an extension of the incubation time from 4 to 12 h, the coefficient decreased from 0.61 to 0.28. This observation indicates that lysosomal escape could be achieved after the endocytosis of PPAH NPs and that the lysosomal escape ability was enhanced with prolonged incubation time.

Subsequently, we proceeded to evaluate their cytotoxicity using the CCK‐8 assay. As depicted in Figure 4C, both PPA and PPAH NPs demonstrated excellent biocompatibility, exhibiting cell viability over 95% at a concentration of 125 µg mL^−1^. Upon application of the NIR laser, PPAH NPs showed obvious photothermal toxicity to BT474 cells, with a clear correlation observed between the cell survival rate and PPAH NP concentration. The anticancer activity of PPAPH+L surpassed that of PPAH+L and PPA+L at equivalent doses (Figure 4D), highlighting the synergistic effect of photothermal ablation and chemotherapy. Given the inherent photocatalytic activity of PPAPH NPs, we analyzed the intracellular generation of ROS in different treatment groups using a 2′,7′‐dichlorodihydrofluorescein diacetate (DCFH‐DA) probe. The CLSM images revealed significant green fluorescence in the PPAH+L and PPAPH+L groups, whereas the control group treated with PBS showed minimal fluorescence (Figure 4E and Figure S18, Supporting Information). These results confirm the ability of PPAPH to induce ROS production through photo‐enhanced catalytic activity. To further visualize live and dead cells, BT474 cells were stained with Calcein‐AM/propidium iodide (PI) following different treatments for CLSM imaging. The CLSM images showed a certain degree of cell death in both the PPAPH and PPAH+L groups, however, the PPAPH+L treatment induced the highest level of cell death (Figure 4F and Figure S19, Supporting Information). This conclusion was further supported by trypan blue staining (Figure S20, Supporting Information) and flow cytometric apoptosis results (Figure 4G and Figure S21, Supporting Information). These results suggested that the combined chemo‐phototherapeutic effect of PPAPH NPs significantly enhanced their cytotoxicity.

**Figure 5:**
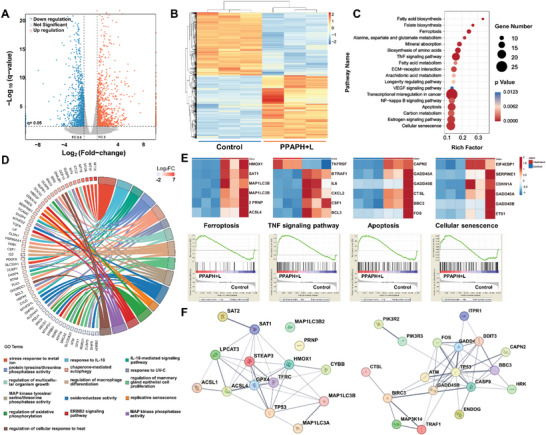
A) Volcano plot of differentially regulated genes between the control and PPAPH+L group. B) Heat map of expressed genes involved in the synergistically therapeutic progress. C) KEGG and D) GO pathway enrichment analysis of the identified differentially expressed genes. E) Heatmaps and GSEA enrichment analysis of differentially expressed genes associated with ferroptosis, TNF signaling pathway, apoptosis, and cellular senescence. F) Functional protein interaction network analysis of the ferroptosis and apoptosis signaling pathway.

To probe into the potential mechanism underlying the synergistic therapeutic effects of chemo‐phototherapy at the genetic level, BT474 cells treated with PPAPH+L were collected for RNA sequencing.^[^ ^28^, ^29^ ^]^ The control group was treated with PBS without 808 nm irradiation. As depicted in the volcano plot (**Figure** **5**A), with FC > 2 and q < 0.05, there were 1362 differentially expressed genes between the control and experimental groups, including 680 upregulated genes and 682 downregulated genes. Differential genes were then used for clustering to draw a heat map (Figure 5B), which confirmed the distinct genetic disparities between the two groups. The Kyoto Encyclopedia of Genes and Genomes (KEGG) and Gene Ontology (GO) databases were used to elucidate the biological functions of these genes in cells. According to the bubble chart of KEGG analysis (Figure 5C), differentially expressed genes were enriched in multiple amino acid and fatty acid metabolism, pro‐inflammation, cell cycle, and cell death pathways, including ferroptosis and apoptosis. GO analysis also demonstrated that abnormal genes were enriched in biological processes related to oxidative stress and cell apoptosis (Figure 5D). In addition, we observed the downregulation of genes involved in the ERBB2, EGFR, and MAPK signaling pathways after PPAPH+L treatment, which validated the inhibitory effect on HER2 (Figure S22, Supporting Information).

Furthermore, enrichment of differentially expressed genes in gene set units mainly associated with cell death was assessed using gene set enrichment analysis (Figure 5E). The results indicated that treatment with PPAPH+L induced the upregulation of HMOX1,^[^ ^30^ ^]^ SAT1,^[^ ^31^ ^]^ and ASCL4, validating that the ROS generated by PPAPH+L disturbed the REDOX and lipid balance in cells, resulting in cell ferroptosis. Additionally, PPAPH+L also elicited genetic alterations involving TNFRSF1B (TNFR2), TRAF1, IL‐6,^[^ ^32^ ^]^ CXCL2,^[^ ^33^ ^]^ CSF1, connected to the TNF signaling pathway, and NF‐κB signaling pathway, which could promote immune activation and immunogenic cell death (ICD). Genes related to cell apoptosis and cellular senescence, such as CAPN2, GADD45A, GADD45B, CTSL, EIF4EBP1 (4E‐BP1),^[^ ^34^ ^]^ SERPINE1, and CDKN1A^[^ ^35^ ^]^ were also upregulated after PPAPH+L treatment. This indicated that PPAPH+L could efficiently enhance the production of ROS and disrupt the oxidation‐reduction process in the cytoplasm, thereby inducing transcriptional misregulation, ferroptosis, and apoptosis in cancer cells. Additionally, protein‐protein interaction (PPI) network analysis was performed based on significantly altered ferroptosis and apoptosis signaling pathways (Figure 5F). These findings suggest that PPAPH‐enabled synergistic chemo‐phototherapy can result in highly efficient cancer cell destruction and tumor inhibition.

**Figure 6:**
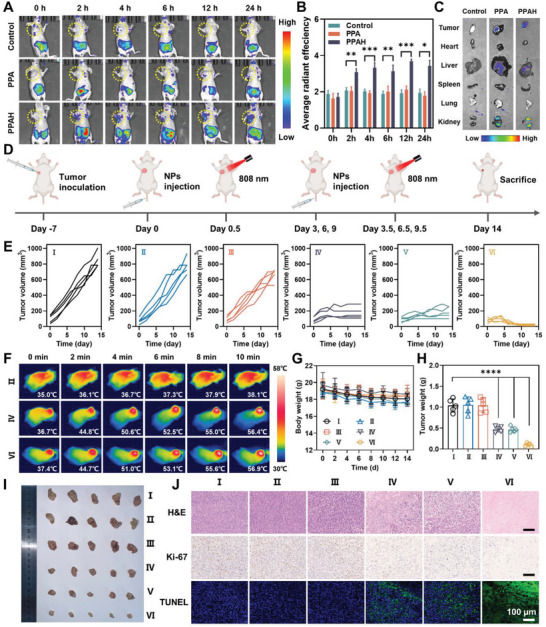
A) In vivo fluorescence imaging at different time points after intravenous injection of PBS, Cy5.5‐labeled PPA NPs, and Cy5.5‐labeled PPAH NPs. B) In vivo fluorescence intensity of control, Cy5.5‐labeled PPA NPs and Cy5.5‐labeled PPAH NPs (*n* = 5, mean ± s.d.). (C) In vitro fluorescence imaging of major organs after different treatments. D) Schematic illustration of in vivo therapeutic procedure on BT474 tumor‐bearing mice. E) The tumor volume of each mouse in each group changed during treatment. F) Infrared thermal images at the tumor sites of mice under 808 nm laser irradiation. G) Average body weight change curves of mice after different treatments (*n* = 5, mean ± s.d.). H) Average tumor mass after 14 days of treatment (*n* = 5, mean ± s.d.). The *p* values were calculated by one‐way ANOVA, ****p < 0.0001, ***p < 0.001, **p < 0.01, and *p < 0.05. I) Photographs of tumors collected from mice after 14 days of treatment. J) Histological images of tumor slices were collected from the mice on day 14. Scale bar: 100 µm. (I, Control; II, Laser; III, PPAH; IV, PPAH+L; V, PPAPH; VI, PPAPH+L).

Before in vivo anti‐tumor therapy, we validated the targeting ability of PPAH NPs. PBS, PPA NPs (non‐targeted), and PPAH NPs (targeted) were injected into the tail vein of mice, followed by in vivo fluorescence imaging. The PPAH NP group exhibited significant fluorescent signals at the tumor site from 0 to 24 h post‐injection compared to the PBS and PPA NP groups. The fluorescence peaked at 12 h (**Figure** **6**A,B). At 24 h post‐injection, the tumors and major organs were harvested for examination. Notably, the fluorescence signal from the tumors in the PPAH NPs group was significantly stronger than that in the non‐targeted group (Figure 6C and Figure S23, Supporting Information). These findings indicated that PPAH NPs possessed the capability to actively target HER2 overexpressing breast cancer cells, exhibiting enhanced accumulation at the tumor site and prolonged retention.

Subsequently, we used BT474 cells to establish a breast tumor model for assessing the efficacy of PPAPH NPs in inhibiting tumor growth (Figure 6D). Mice‐bearing tumors were randomly allocated into six groups and treated with PBS, PBS+L, PPAH, PPAH+L, PPAPH, and PPAPH+L formulations. In a typical protocol, mice in groups receiving laser irradiation were exposed to an 808 nm laser (1.0 W cm^−2^) 12 h after intravenous injection of the drug formulation, and the tumor site temperature was measured with a thermal imager. With the accumulation of NPs at the tumor site, the temperature at the tumor rapidly increased to 50.0 °C within 4 min, whereas the temperature in the control group only increased by 3.1 °C (Figure 6F). This significant temperature increase can be attributed to the efficient photothermal conversion capability of PPAH NPs. Throughout the treatment, tumor growth was diligently monitored (Figure 6E). Post‐treatment, breast tumors were resected for weighing and imaging (Figure 6H,I). Among these groups, PPAPH+L exhibited the most effective inhibition of tumor growth, with the smallest tumor volume and weight. The excised tumors were further stained with hematoxylin and eosin (H&E), Ki‐67 antibody, and terminal deoxynucleotidyl transferase dUTP nick end labeling (TUNEL) (Figure 6J). The PPAPH+L group showed more pronounced cell necrosis and apoptosis, and the proliferation rate was inhibited compared to the other groups. This can be attributed to the synergistic effects of effective tumor targeting and multimodal combination therapy.

**Figure 7:**
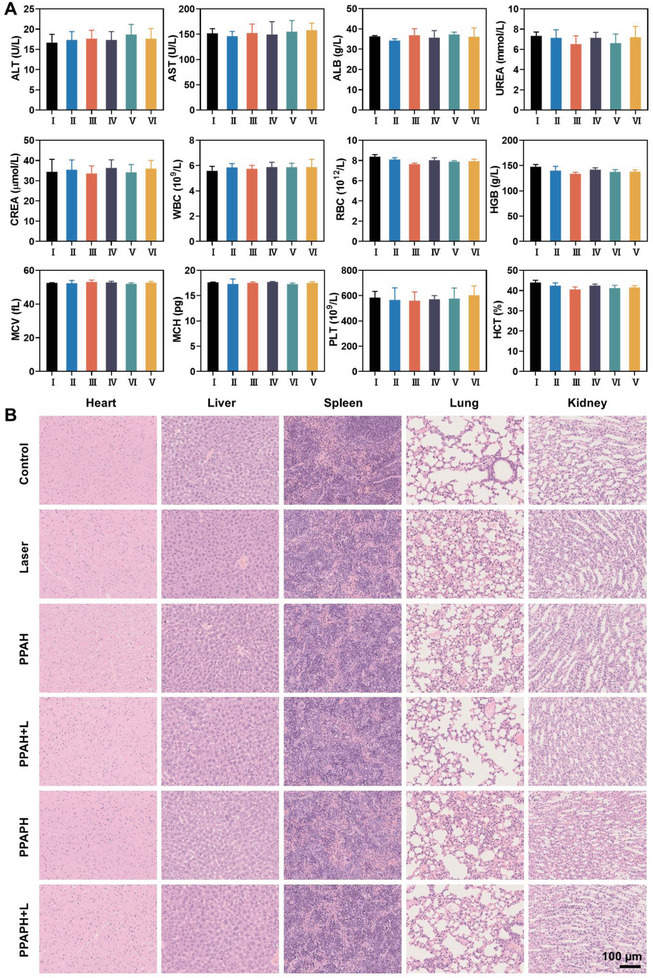
A) Blood biochemical tests (alanine aminotransferase (ALT), aspartate aminotransferase (AST), albumin (ALB), urea nitrogen (UREA), creatinine (CREA)) and blood routine of these mice (white blood cells (WBC), red blood cells (RBC), hemoglobin (HGB), mean corpuscular volume (MCV), mean corpuscular hemoglobin (MCH), platelets (PLT), hematocrit (HCT)) (*n* = 5, mean ± s.d.). B) H&E staining of tumor tissues after different treatments. Scale bar: 100 µm.

In terms of the toxicity profile of the nanoparticles, the mice did not exhibit noticeable body weight loss during the treatment period (Figure 6G). Moreover, blood routine and biochemical tests showed no significant aberrant indices compared to the control group after intravenous injection of PPAPH NPs (**Figure** **7**A), indicating that the nanoparticles have good blood compatibility. The major organs of the mice, including heart, liver, spleen, lungs, and kidneys, were processed using H&E staining (Figure 7B). Compared with the negative control, the effects of PPAPH NPs on tissue structure and integrity were negligible. This suggests that nanoparticles possess good biocompatibility for in vivo applications.

## 3 Conclusion

In conclusion, this study presents a tumor‐targeted multifunctional nanotherapeutic platform (PPAPH) for the synergistic chemo‐phototherapy treatment of HER2‐positive breast cancer. PPAPH employs AuAg HNSs as substrates, which can be efficiently loaded with PYR and shows pH/NIR light dual‐stimuli‐responsive drug release properties. Notably, the intrinsic properties of the AuAg HNSs enable them to serve as both effective photothermal and photocatalytic agents. Under 808 nm laser irradiation, the PPAPH NPs exhibited a significantly high PCE of 34% while maintaining desirable photothermal stability. Photoexcitation concurrently generates hot electrons, thereby augmenting the catalytic activity of the nanoparticles. Following treatment with PPAPH NPs, the HER2 signaling pathway was suppressed and oxidative stress was amplified with the assistance of light, resulting in ferroptosis and apoptosis. The robust anticancer efficacy of PPAPH NPs has been validated as they could suppress tumor growth and reduce tumor size, particularly under NIR light irradiation, with minimal impact on healthy tissues. This highlights the promising prospects of these nanoparticles for future clinical applications. Moreover, it paves the way for the use of this multimodal hybrid nanoplatform in the treatment of other HER2‐positive cancers.

## 4 Experimental Section

### Synthesis of Gold‐Silver Hollow Nanoshells (AuAg HNSs)

Ag NPs (10 mL) were combined with 100 mL of the K‐gold solution and stirred for ≈5 h, resulting in a blue‐colored solution. The solution was centrifuged at 8000 rpm for 15 min, followed by the removal of the supernatant. Subsequently, the residue was redispersed in 15 mL ultrapure water. Please note that no attempt was made to recover any residue stuck to the side of the centrifuge tube via sonication. Any such residue was discarded. Samples with different absorption wavelengths were prepared by varying the volume ratio of Ag NPs and K‐gold solution. Specifically, different volumes of Ag NPs (1.75, 1.5, 1.25, 1.0, and 0.75 mL) were introduced to 10 mL of the K‐gold solution under stirring. The solution color rapidly changed from yellow to blue and was monitored by UV–vis spectroscopy.

### Synthesis of Herceptin Functionalized AuAg HNSs

HCT activation was achieved using EDC/NHS chemistry, and PEG‐PEI‐AuAg was functionalized with the activated HCT. First, EDC (8 mg) and NHS (4 mg) were dissolved in 2 mL PBS, and allowed to stand for 30 min. Subsequently, 2.0 mL of HCT solution (0.2 mg mL^−1^) was added to the mixture and magnetically stirred. After 2 h, PEG‐PEI‐AuAg (3 mL) was introduced into the reaction solution, followed by stirring at 10 °C for 12 h. Finally, excess HCT, EDC, and NHS were removed by centrifugation, and the obtained PPAH were redispersed in 6 mL of ultrapure water (2 mg mL^−1^) and stored at 4 °C for later use.

### Pyrotinib (PYR) Loading

A solution of 3 mg PYR dissolved in 200 µL of DMSO was added dropwise into 3 mL of AuAg HNSs and stirred for 12 h. Then, the LA‐PEI aqueous solution was added and stirring was continued. After 12 h, the mixture was centrifuged and washed with water. SH‐PEG and HCT were modified following steps 4.3 and 4.4. The supernatant and precipitate were subsequently separated by centrifugation, and the obtained PPAPH NPs were redispersed in 6 mL of ultrapure water (2 mg mL^−1^) and stored at 4 °C. PYR loading was determined by measuring the UV–vis absorption spectra. The EE and LC were calculated according to the standard curve and the following equations.

EE (%, w/w) = m (loading PYR)/m (total PYR) × 100

LC (%, w/w) = m (loading PYR)/m (total NPs + loading PYR) × 100

### PYR Release

PPAPH solution (1 mg mL^−1^, 1 mL) was added to the dialysis bag. The dialysis bags were then immersed in 5 mL of PBS with different pH (5.5, 6.5, and 7.4) and stored at 37 °C under constant temperature and shaking at 200 r min^−1^. At selected time points (1, 2, 4, 6, 18, 30, 42, 54, and 72 h), 1 mL of the solution was taken out and replaced with 1 mL of fresh PBS. The PYR content was measured using UV–vis absorption spectra. To investigate the laser‐driven drug release behavior of PPAPH NPs, PPAPH was subjected to an 808 nm laser irradiation (1.0 W cm^−2^) for 10 min at selected time points (2, 4, 6, and 8 h). The PBS solution was collected for absorption spectrum measurement to determine the amount of released PYR.

### Cellular Uptake

BT474 cells were inoculated into confocal plates at 1 × 10^5^ cells per plate. After incubation for 24 h, Cy5.5‐labeled PPAH NPs (100 µg mL^−1^) were added. After co‐culture for 1, 2, 4, or 6 h, the cells were washed three times with cold PBS. Fluorescence images of cancer cells were captured using CLSM.

For in vitro targeting studies, MCF‐7, MDA‐MB‐231, and BT474 cells were inoculated into confocal plates at 1 × 10^5^ cells per plate. After 24 h of incubation, Cy5.5‐labeled PPAH NPs (100 µg mL^−1^) were added. After 6 h of co‐culture, the cells were washed with cold PBS. Fluorescence images of the cancer cells were observed using CLSM.

### Lysosome Escape

BT474 cells were seeded in confocal plates at 1 × 10^5^ cells per plate and incubated at 37 °C for 24 h. Thereafter, the Cy5.5‐labeled PPAH NPs were added and incubated at 37 °C for predetermined periods. The lysosome was labeled with Lysotracker red before CLSM observations.

### Cellular Reactive Oxygen Species Detection

BT474 cells were seeded at 1 × 10^5^ cells per plate in confocal plates and then treated with PBS, PPA, PPAH, and PPAPH (100 µg mL^−1^), respectively. 12 h later, NIR laser (1.0 W cm^−2^, 10 min) stimulation was applied and incubation was continued for 12 h. After staining with DCFH‐DA for 30 min, intracellular ROS levels were observed using CLSM.

### Live‐Dead Cell Staining

BT474 cells (1 × 10^5^ cells per plate) were inoculated into confocal plates and treated with PBS, PPA, PPAH, and PPAPH (100 µg mL^−1^). 12 h later, NIR laser (1.0 W cm^−2^, 10 min) stimulation was applied. After 12 h of continued incubation, the cells were stained with a Calcein‐AM/PI working solution for 20 min for further CLSM observation.

### Flow Cytometric Analysis

BT474 cells (1 × 10^5^ cells per plate) were inoculated into 6 well‐plates and treated with PBS, PPA, PPAH, and PPAPH (100 µg mL^−1^). 12 h later, NIR laser (1.0 W cm^−2^, 10 min) stimulation was applied. After 12 h of continued incubation, the cells were suspended and stained with propidium iodide and FITC‐Annexin V reagent for flow cytometric analysis using a FACS Calibur flow cytometer.

### Animals

5‐week‐old female BALB/c mice (19–22 g) were purchased from Beijing Huafukang Biotechnology Co., Ltd. All animal procedures were approved by the Committee on the Ethics of Animal Experiments of the Second Hospital of Shandong University (Jinan, China, Permit No. KYLL‐2021(KJ)A‐0401). BT474 cells (2 × 10^7^) were inoculated on the right armpit of mice. The experiments were performed after the tumor volume reached ≈60 mm^3^.

### In Vivo Targeting Studies

Tumor‐bearing nude mice were separated into control, targeted, and non‐targeted groups and subsequently injected with PBS, Cy5.5‐labeled PPAH NPs, and Cy5.5‐labeled PPA NPs (15 mg kg^−1^) via intravenous administration. After injection of the materials, optical fluorescence imaging was performed using a 3D optical live imaging system (IVIS Spectrum, USA) at the indicated time points. The mice were sacrificed 72 h after the intra‐tumor injection of drugs. Tumors and organs (liver, heart, lungs, spleen, and kidneys) were dissected for in vivo imaging.

### Anti‐Tumor Effect and Biosafety Evaluation

The mice were randomly separated into six groups and intravenously injected with different formulations: (I) PBS, (II) PBS+L, (III) PPAH, (IV) PPAH+L, (V) PPAPH, and (VI) PPAPH+L at a dose of 15 mg kg^−1^. For groups (II), (IV) and (VI), the tumors were irradiated with an 808 nm laser (1.0 W cm^−2^) for 10 min. The tumor volumes and body weights were recorded every 2 days and the volume of tumor were calculated as follows: tumor volume = length × width^2^/2. After treatment for 14 days, these mice were sacrificed, and their whole blood, blood serum, and major organs and tumors were collected for complete blood panel analysis, blood chemistry, and histology examination, respectively.

### Statistical Analysis

GraphPad Prism 8 software was used to analyze the data. All the results in this work were presented as mean ± s.d. and represented a minimum of three independent experiments. Statistical analyses were performed by Student's *t*‐test or one‐way ANOVA. *p* < 0.05 was acknowledged as statistically significant.

## Conflict of Interest

The authors declare no conflict of interest.

## Supporting information

Supporting Information

## Data Availability

The data that support the findings of this study are available from the corresponding author upon reasonable request.
