# Supplementary material for: A Multifunctional Bimetallic Nanoplatform for Synergic Local Hyperthermia and Chemotherapy Targeting HER2‐Positive Breast Cancer
Source: Adv Sci (Weinh). 2024 Feb 21;11(16):2308316. doi: 10.1002/advs.202308316 (PMC11040336; doi:10.1002/advs.202308316)
Supplement: Supplementary file 1 — Supporting Information [file ADVS-11-2308316-s001.pdf]

ADVANCED SCIENCE
Open Access

Supporting Information

for *Adv. Sci.*, DOI 10.1002/advs.202308316

A Multifunctional Bimetallic Nanoplatform for Synergic Local Hyperthermia and Chemotherapy Targeting HER2-Positive Breast Cancer

Li Zhao, Fei Chang, Yao Tong, Jiawei Yin, Jiawen Xu, Hui Li, Lutao Du and Yanyan Jiang**

Supporting Information

A Multifunctional Bimetallic Nanoplatform for Synergic Local Hyperthermia and Chemotherapy Targeting HER2-Positive Breast Cancer

Li Zhao[a, #], Fei Chang[b, #], Yao Tong[b], Jiawei Yin[b], Jiawen Xu[c], Hui Li[a], Lutao Du[b, d, e, f, *], Yanyan Jiang[a, *]

[a] Liquid-Solid Structural Evolution & Processing of Materials (Ministry of Education), School of Materials Science and Engineering, Shandong University, Jinan, Shandong, 250061, China

[b] The Second Hospital of Shandong University, Jinan, Shandong, 250033, China

[c] Department of Pathology, Shandong Provincial Hospital affiliated to Shandong First Medical University, Jinan, Shandong, 250021, China

[d] Department of Clinical Laboratory, Qilu Hospital of Shandong University, Jinan, Shandong, 250012, China

[e] Shandong Provincial Key Laboratory of Innovation Technology in Laboratory Medicine, Jinan, Shandong, 250033, China;

[f] Shandong Provincial Clinical Medicine Research Center for Clinical Laboratory, Jinan, Shandong, 250033, China

* Corresponding author.

E-mail addresses: lutaodu@sdu.edu.cn (L. T. Du); yanyan.jiang@sdu.edu.cn (Y. Y. Jiang)

[#] L. Zhao and F. Chang contributed equally to this work.

Supplementary Methods

Materials

Gold (III) chloride trihydrate ($HAuCl_4 \cdot 3H_2O$, 99%), potassium carbonate (K_2CO_3), sodium citrate dihydrate, potassium iodide (KI), lipoic acid (LA), branched polyethyleneimine (PEI), 1-(3-Dimethylaminopropyl)-3ethylcarbodiimide hydrochloride (EDC) and N-hydroxysuccinimide (NHS) were purchased from Shanghai Macklin Biochemical Co. Ltd. Silver nitrate ($AgNO_3$) and ascorbic acid were purchased from Sinopharm Chemical Reagent Co., Ltd. SH-PEG (MW=5000) was purchased from Shanxi Kairui Aosheng Biotechnology Co., Ltd. PYR was purchased from Jiangsu Hengrui Pharmaceutical Co., Ltd. HCT was purchased from Roche Pharma (Basel, Switzerland). Cell culture reagents, including penicillin-streptomycin, DMEM medium, fetal bovine serum, trypsin-EDTA, Cell Counting Kit-8 (CCK-8), trypan blue, 2',7'-dichlorofluorescein diacetate (DCFH-DA), Calcein-AM (AM)/propidium iodide (PI) and Annexin V-FITC/PI Apoptosis Detection Kit were purchased from Gibco (Shanghai, China). All chemicals were used as received without further purification. Ultrapure water (Millipore Milli-Q grade) with a resistivity of 18.2 MΩ was used in all the experiments.

Characterization

The UV-vis-NIR absorption spectra were measured using a SPECORD 200 PLUS spectrophotometer (Analytik Jena AG). Transmission electron microscopy (TEM) images were obtained using an HT-7700 system (JEOL, Japan). High-angle annular dark-field scanning

transmission electron microscopy (HAADF-STEM) and energy-dispersive X-ray spectroscopy (EDS) elemental mapping were performed using a JEM-2200FS electron microscope operating at 200 kV. Powder X-ray diffraction (XRD) patterns were recorded on D8 DISCOVER with Cu Kα radiation (λ=1.542 Å) operating at 50 kV and 300 mA. Fourier transform infrared spectroscopy (FTIR) was recorded on a spectrometer (Nicolet 6700) by the KBr tablet method, and the spectra were scanned in the range of 400-4000 cm^{-1} at a resolution of 4 cm^{-1}. The particle size distribution and zeta potential were measured by dynamic light scattering (DLS) using Malvern Zetasizer Nano ZS (Malvern Instruments Ltd., Worcestershire, UK). An infrared laser (LSR808NL-3W-FC, Ningbo Yuanxin Optoelectronic Technology Co., Ltd.) was used to test the photothermal properties of the material, and the temperature change in the material under laser irradiation was recorded using an infrared imager (FLIRE8XT, Estonia). A scanning confocal fluorescence imaging microscope (CLSM800, Carl Zeiss, Germany) was used to observe cellular uptake. The cytotoxicity of the materials was determined using an enzyme-labeled instrument (Infinite M200 Pro, Switzerland). Cell apoptosis was assessed using flow cytometry. The fluorescence images *in vivo* were obtained using a fluorescence imaging system (IVIS Spectrum, PerkinElmer).

Synthesis of Silver Nanoparticles (Ag NPs)

The first step involved heating 95 mL of water in a 250 mL round-bottom flask to 100 °C. 1 mL of ascorbic acid solution (5 mM) was added to the flask, which was heated to 120 °C. Concurrently, 0.0167 g $AgNO_3$ was dissolved in 2 mL of water, followed by the addition of 2 mL of 1% sodium citrate solution and 50 µL of 7 µM KI solution, which was sonicated for 10 min. Subsequently, the mixed solution was added to the flask when it stabilized at 120 °C, followed by refluxing at 120 °C for 1 h. A brownish-yellow solution formed, consistent with the presence of

Ag NPs. The solution was allowed to cool to room temperature and centrifuged at 8000 rpm for 15 min. After removing the supernatant, the Ag NPs were transferred to a fresh centrifuge tube, redispersed in ultrapure water (12.5 mL), and refrigerated. Please note that no attempt was made to recover any residue stuck to the side of the centrifuge tube via sonication. Any such residue was discarded.

Synthesis of K-gold Solution

0.025 g of K_2CO_3 was added to 100 mL of ultrapure water, which was then infused with varying amounts of 1% $HAuCl_4 \cdot 3H_2O$ solution under stirring. The mixture (initially yellow) became colorless after stirring for 30 min. The flask was then covered with aluminum foil to shield it from light and stored overnight in a refrigerator.

Preparation of LA-PEI

PEI (300 mg) was dissolved in water, and the pH was adjusted to neutral using 1.0 M HCl to obtain a PEI solution. LA (82.4 mg) was completely dissolved in acetonitrile (20 mL) and EDC (191.7 mg) and NHS (115.1 mg) were added. The above solution was mixed with the PEI solution, stirred for 24 h, dialyzed in a dialysis bag (MW=7000 Da) for 48 h, and then freeze-dried with a freeze dryer to obtain the LA-PEI sample.

Preparation of PEI-AuAg

3 mL of AuAg HNSs were added to the reaction flask, then 4 mL of LA-PEI (20 mg) aqueous solution was added and stirred at room temperature for 12 h. Finally, excess LA-PEI was removed by centrifugation, and the resulting PEI-AuAg was redispersed in 3 mL of ultrapure water.

Preparation of PEG-PEI-AuAg

1 mL of SH-PEG (5 mg) was added to 3 mL of the PEI-AuAg solution and stirred for 12 h.

Excess SH-PEG was then removed by centrifugation, and the resulting PEG-PEI-AuAg was redispersed in 3 mL of ultrapure water.

Photothermal Effects and Photothermal Conversion Efficiency

To evaluate the photothermal performances of PPAH NPs, 500 μL of PPAH aqueous solution with different concentrations (62.5, 125, 250, and 500 μg/mL) were irradiated by 808 nm laser at a power density of 1.0 W/cm^2 for 10 min. Temperature changes were monitored using an infrared camera and analyzed using FLIR software. Similarly, the PPAH aqueous solution (500 μL, 125 μg/mL) was exposed to an 808 nm laser at different power densities (0.5, 1.0, 1.5, and 2.0 W/cm^2) for 10 min, and its temperature changes were also monitored. For the photothermal stability test, the PPAH aqueous solution was subjected to five on/off cycles of 10 min each under a laser at 1.0 W/cm^2. The photothermal conversion efficiency (η) was calculated by the following equations:

$$\eta = \frac{hS(T_{max} - T_{surr}) - Q_s}{I(1 - 10^{-A})}$$

where h, S, T, and Q_s denote the heat-transfer coefficient, tube surface area, solution temperature, and heat associated with the light absorbance of the tube and water, respectively. I and A represent the laser power and absorbance of the solution at 1064 nm, respectively.

To calculate hS, a parameter τ (the time constant) was introduced, which can be defined as:

$$\tau_s = \frac{m_D C_D}{hS}$$

where m_D and C_D are constants representing the mass and heat capacities of the solvent (ultrapure water), respectively. To determine the value of τ_s, the relevant parameters in the cooling curve were linearly fitted using the following equation:

$$t = -\tau_s \, ln(\theta) = -\tau_s \ln(\frac{\mathrm{T_{amb}} - \mathrm{T}}{\mathrm{T_{amb}} - \mathrm{T_{max}}})$$

where T_{amb} and T_{max} represent the room temperature and maximum temperature during

irradiation, respectively.

Photocatalytic Performance

The generation of •OH, with and without light, was assessed using the classical TMB assay. PPAH (1 mg/mL, 200 μL) and H_2O_2 (20 mM, 200 μL) were introduced into 1.5 mL of PBS buffer (0.1 M, pH=6.5). Subsequently, TMB (20 mM, 100 μL) was added to the above mixture with and without 808 nm laser irradiation (1.0 W/cm^2). The change in TMB was recorded at one-minute intervals by UV-vis detection.

The generation of $^1O_2$, with and without NIR irradiation, was measured using DPBF as a specific probe. In detail, PPAH (1 mg/mL, 200 μL) was introduced into 1.78 mL of PBS buffer (0.1 M, pH=6.5). Then DPBF (1 mg/mL, 20 μL) was added to the above mixture to initiate the oxidizing reaction of DPBF with and without 808 nm laser irradiation (1.0 W/cm^2). The oxidation process of DPBF was recorded at different time points using UV-vis spectroscopy.

Cell Culture

BT474 cells were cultured as a monolayer in DMEM supplemented with 10% FBS and 1% antibiotic mixture. The culture flasks were maintained in the incubator at 37 °C, under a 5% CO_2 atmosphere with 95% humidity.

Cell Viability Assays

BT474 cells were seeded in 96-well plates at a density of 1×10^4 cells/well and incubated for 24 h. After cell adhesion, cells were co-incubated with materials at various concentrations (0, 25, 50, 75, 100, and 125 μg/mL) for 12 h. Subsequently, the cells were treated with the CCK-8 solution. After incubation for 4 h, the absorbance was measured at 450 nm using a microplate reader. Relative cell viability (%) = (mean OD_{450} of sample/mean OD_{450} of control) × 100.

For the laser-treated groups, cells were treated with materials at different concentrations (0, 25, 50, 75, 100, and 125 μg/mL) for 12 h, and each well was washed with fresh medium and irradiated under 808 nm laser (1.0 W/cm^2) for 10 min. After incubation for 12 h, CCK-8 assays were performed to determine cell viability.

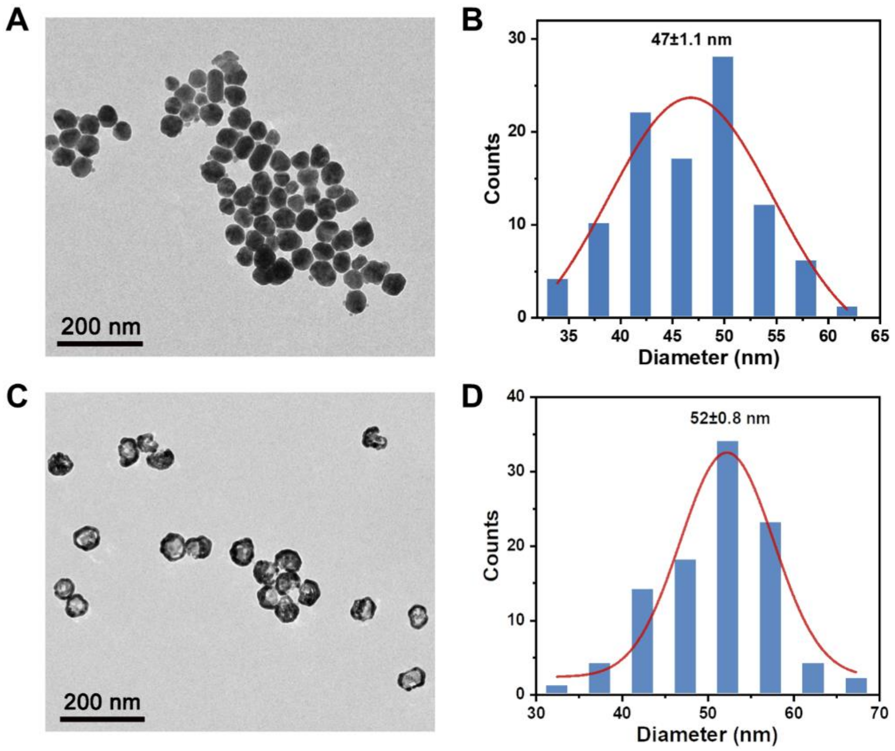

Figure S1 (A) TEM image of Ag NPs. (B) Statistical data of the length of 100 Ag NPs. (C) TEM image of AuAg HNSs. (D) Statistical data of the length of 100 AuAg HNSs.

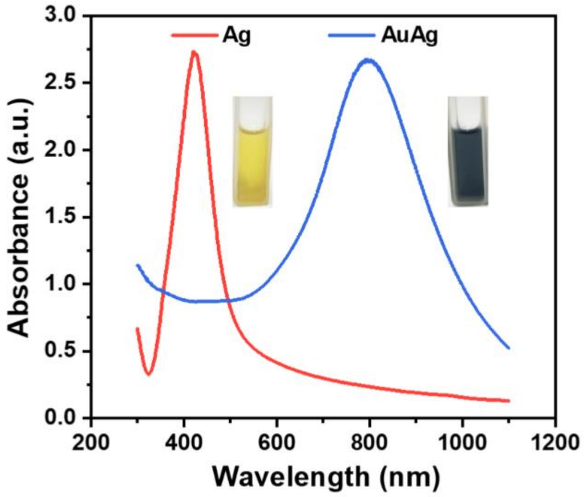

Figure S2 UV-vis-NIR spectra of Ag NPs and AuAg HNSs. Inset: Photographs of Ag NPs and AuAg HNSs.

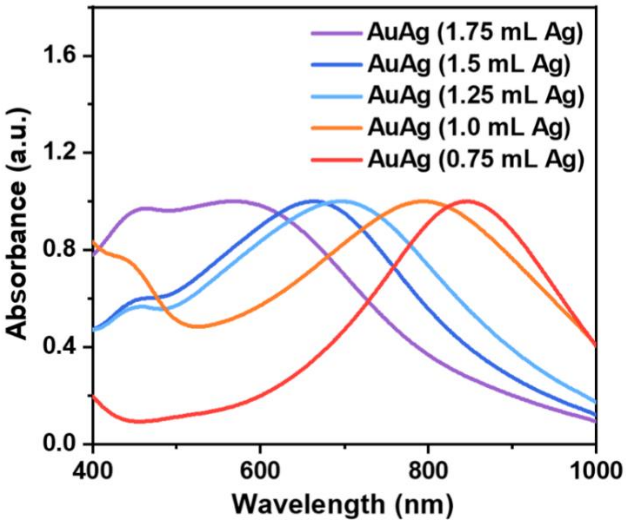

Figure S3 UV-vis-NIR spectra of AuAg HNSs obtained by adding different volumes of Ag NPs (1.75, 1.5, 1.25, 1.0 and 0.75 mL) to 10 mL of K-gold solution.

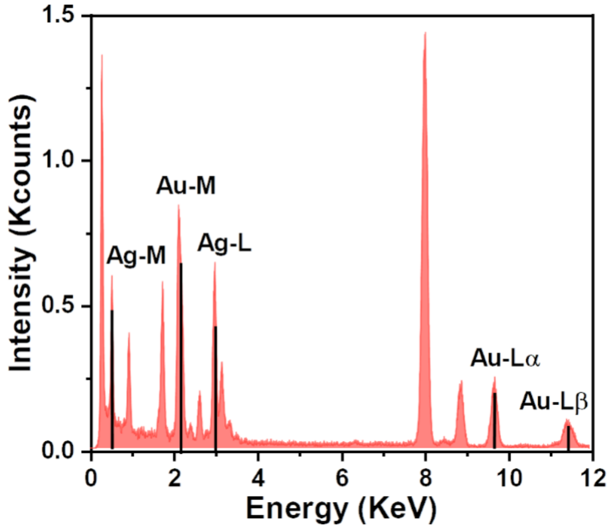

Figure S4 The energy dispersive spectrometer (EDS) elemental mapping of AuAg HNSs.

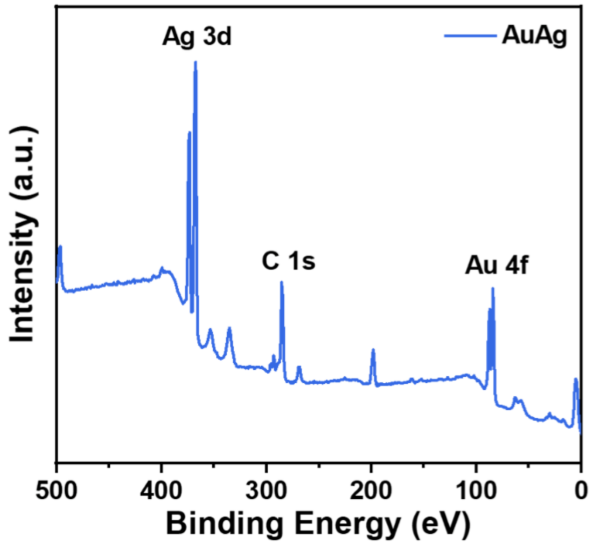

Figure S5 XPS survey spectrum of AuAg HNSs.

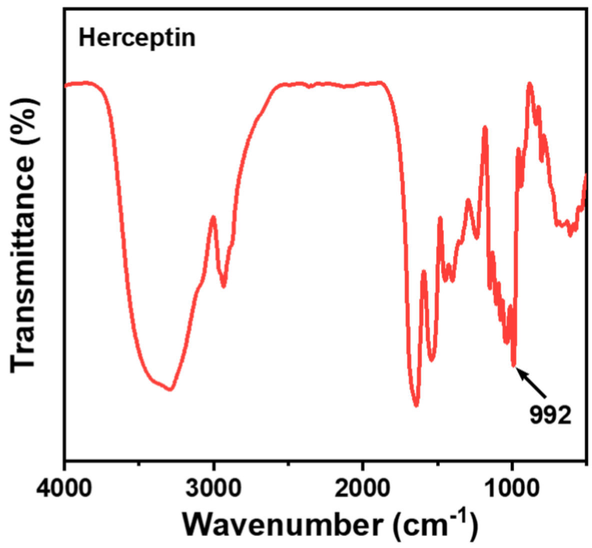

Figure S6 FTIR spectrum of Herceptin.

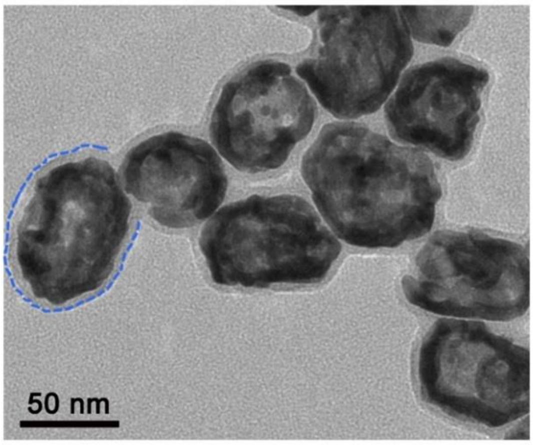

Figure S7 TEM image of PPAH NPs.

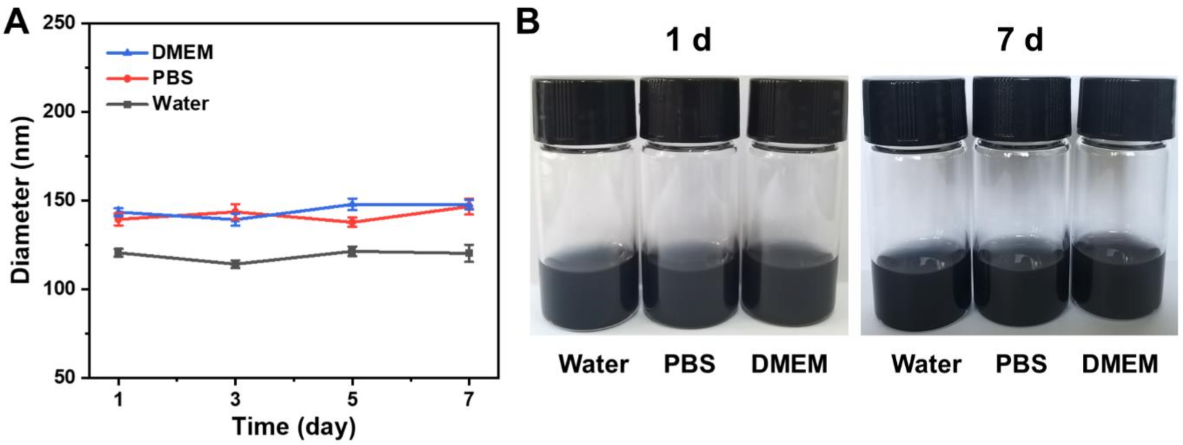

Figure S8 (A) Hydrodynamic size and (B) digital photographs during 7 days dispersed in different physiological mediums (n=3, mean ± s.d.).

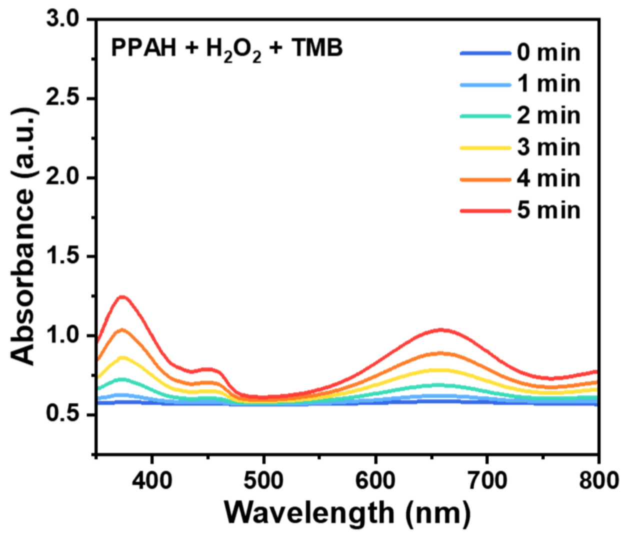

Figure S9 TMB absorbance spectra upon the addition of PPAH NPs and H_2O_2 without irradiation.

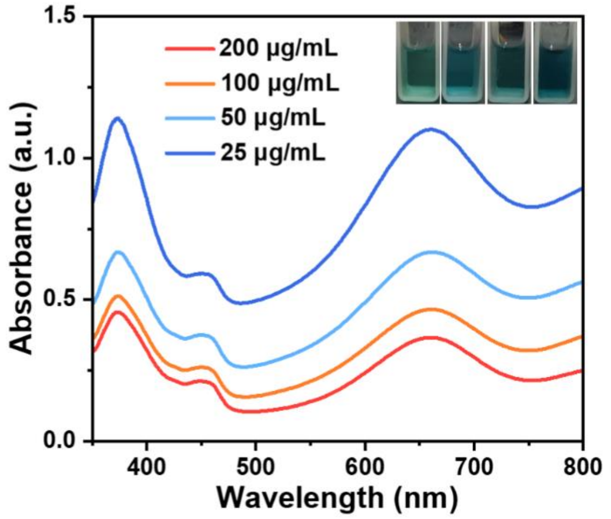

Figure S10 TMB absorbance spectra and photographs upon the addition of different concentrations of PPAH NPs and H_2O_2.

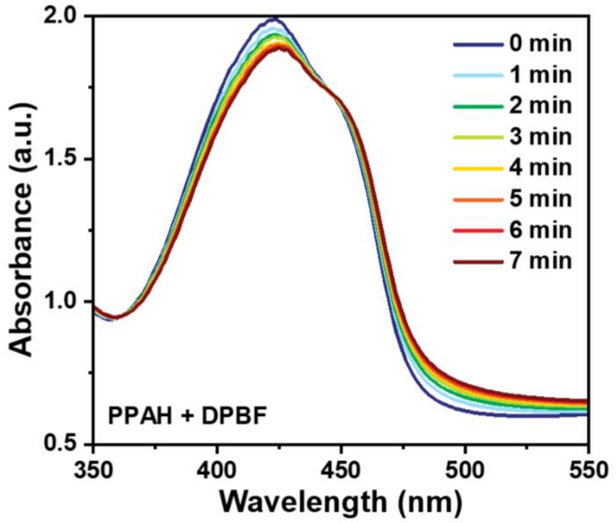

Figure S11 UV-vis absorption spectra of DPBF upon the addition of PPAH NPs without irradiation.

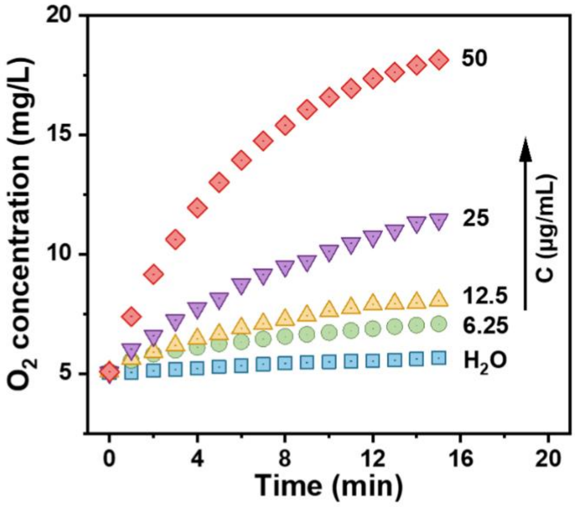

Figure S12 (A) O_2 generation in H_2O_2 solution (10 mM) with different concentrations of PPAH NPs without irradiation.

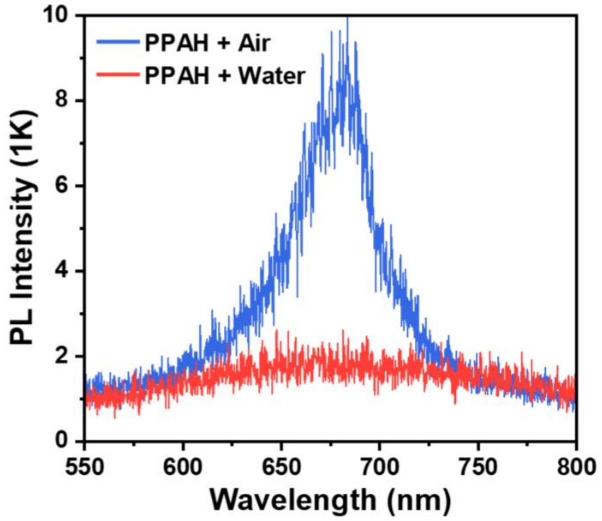

Figure S13 PL intensity of a single PPAH NP in air and immersed in H_2O.

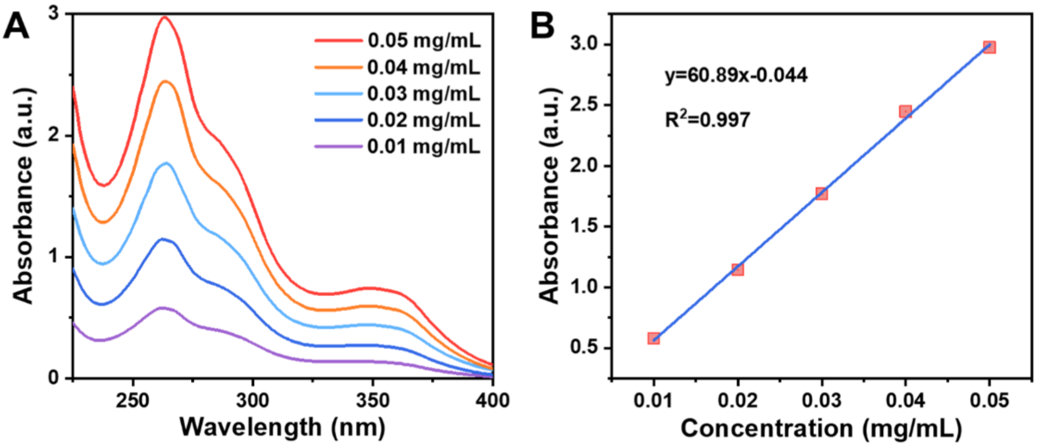

Figure S14 (A) The UV-vis absorption spectra of PYR with different concentrations. (B) The standard curve of the absorbance at 262 nm with the PYR concentration.

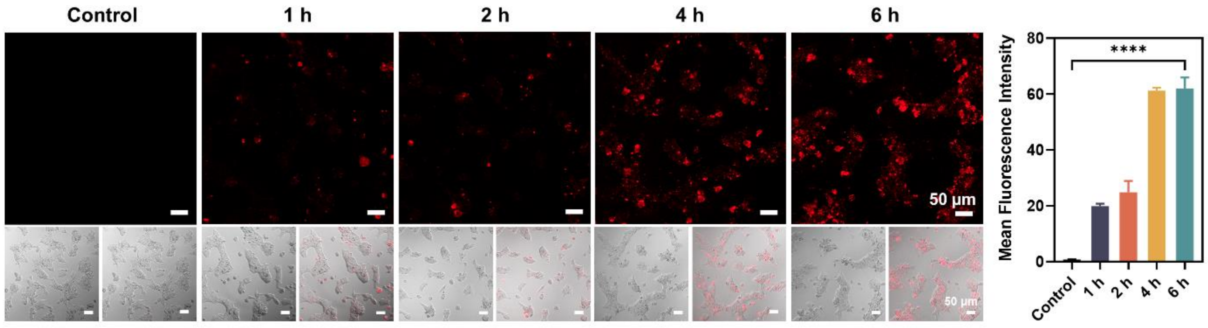

Figure S15 CLSM images of BT474 cells incubated with Cy5.5-labeled PPAH NPs after 1 h, 2 h, 4 h, and 6 h, and the corresponding semiquantitative analysis (n=3, mean ± s.d.). Scale bar: 50 μm.

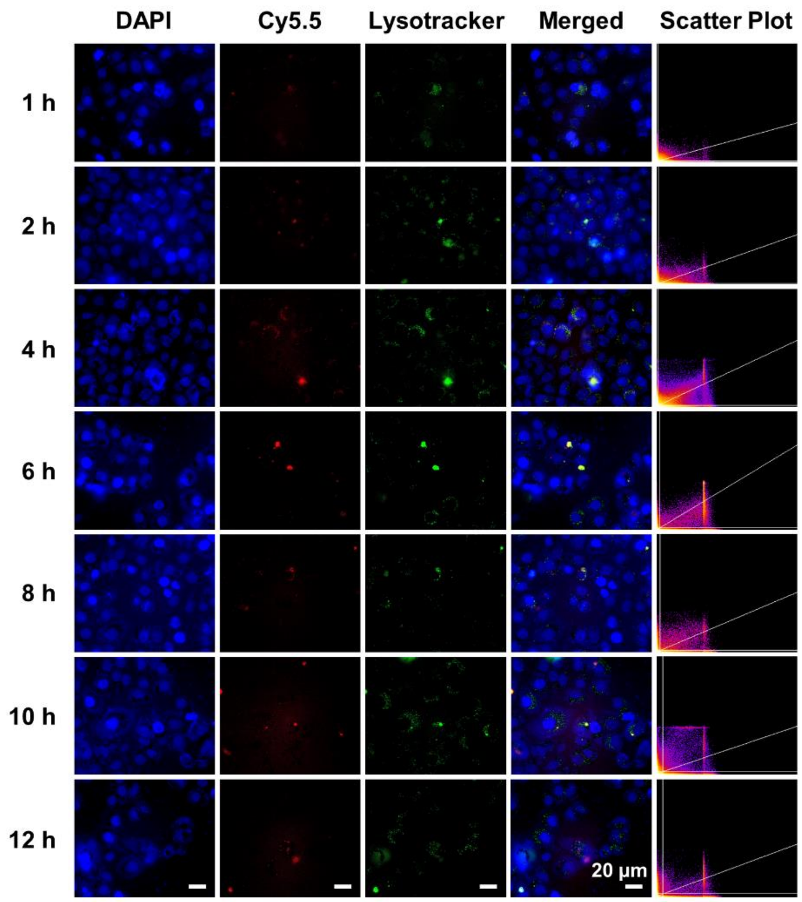

Figure S16 CLSM images of PPAH NPs co-incubated with BT474 cells for 1, 2, 4, 6, 8, 10 and 12 h to assess lysosomal escape capacity. Scale bar: 20 μm.

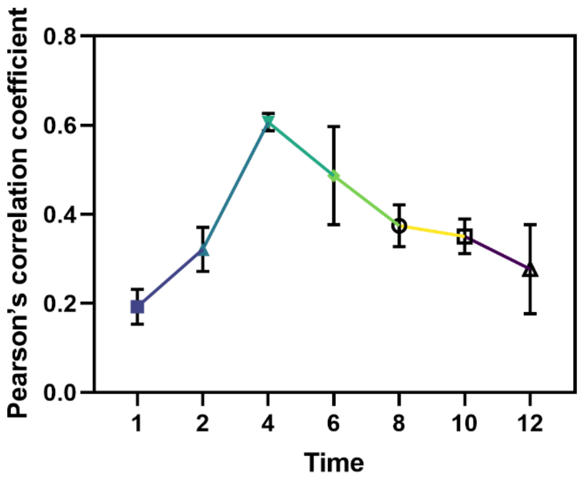

Figure S17 Pearson's correlation coefficient of PPAH NPs colocalized with lysosomes (n=3, mean ± s.d.).

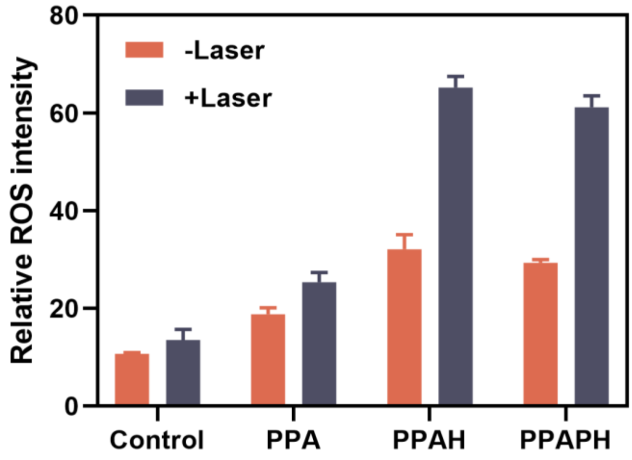

Figure S18 The corresponding quantitative analysis of intracellular ROS levels after different treatments (n=3, mean ± s.d.).

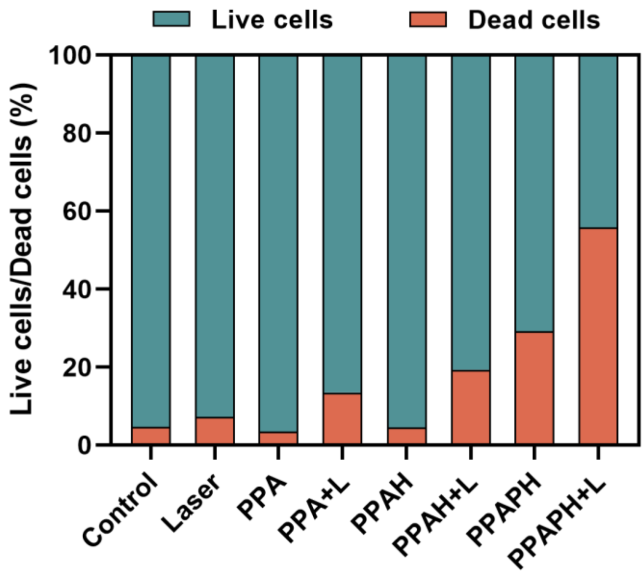

Figure S19 The live/dead cell ratios of BT474 cells stained with Calcein-AM/PI after different treatments.

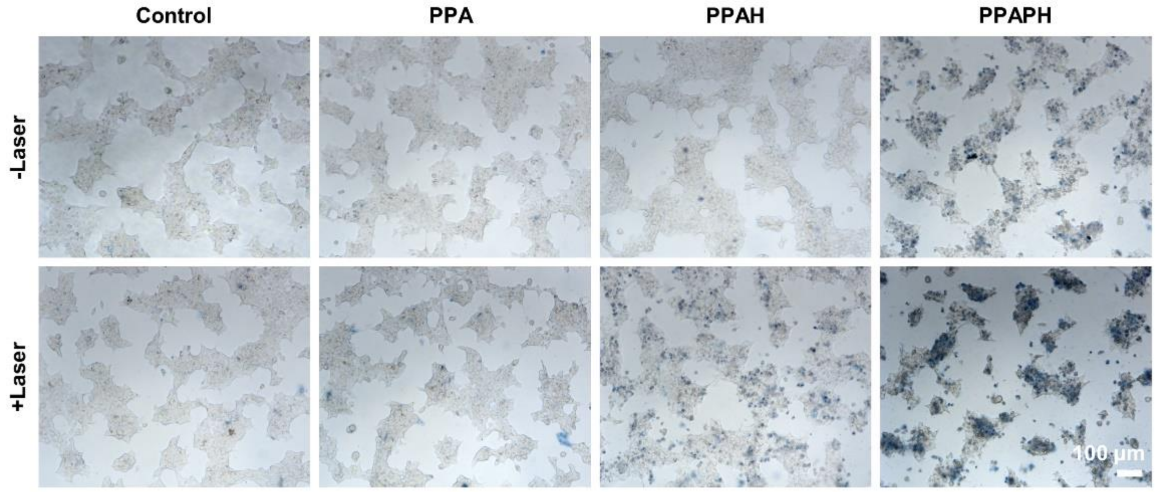

Figure S20 Trypan blue staining of BT474 cells after different treatments. Scale bar: 100 μm.

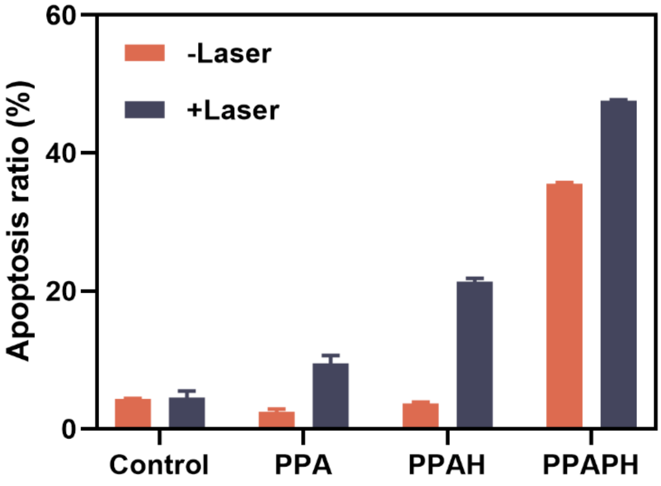

Figure S21 Apoptosis ratio of BT474 cells after different treatments (n=3, mean ± s.d.).

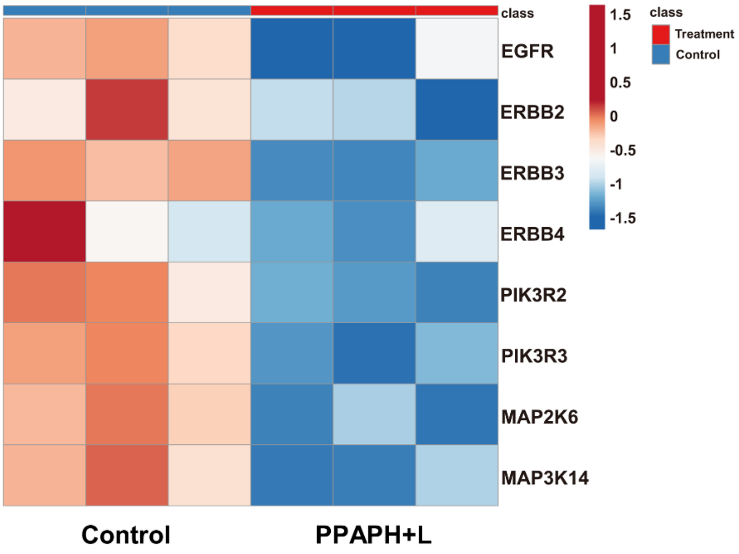

Figure S22 Heat map of differential genes associated with HER2 in BT474 cells after different treatments.

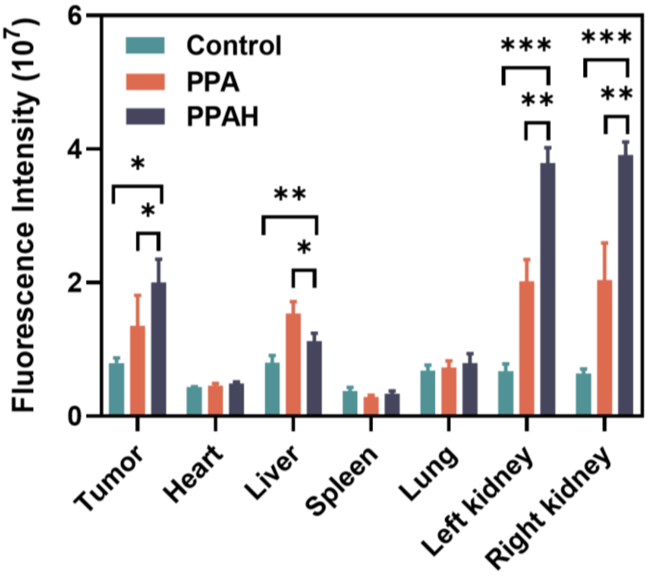

Figure S23 Semiquantitative analysis of the fluorescence intensity in major organs at 24 h (n=5, mean ± s.d.). The p values were calculated by one-way ANOVA, ****$p < 0.0001$, ***$p < 0.001$, **$p < 0.01$, and *$p < 0.05$.
